# Hypoxic exosomes facilitate bladder tumor growth and development through transferring long non-coding RNA-UCA1

**DOI:** 10.1186/s12943-017-0714-8

**Published:** 2017-08-25

**Authors:** Mei Xue, Wei Chen, An Xiang, Ruiqi Wang, He Chen, Jingjing Pan, Huan Pang, Hongli An, Xiang Wang, Huilian Hou, Xu Li

**Affiliations:** 1 Center for Translational Medicine, The First Affiliated Hospital of Xi’an Jiaotong University, Xi’an, People’s Republic of China; 2 Key Laboratory for Tumor Precision Medicine of Shaanxi Province, The First Affiliated Hospital of Xi’an Jiaotong University, Xi’an, People’s Republic of China; 3 Department of Clinical Laboratory, The First Affiliated Hospital of Xi’an Jiaotong University, Xi’an, People’s Republic of China; 4 Department of Pharmacology, Fourth Military Medical University, Xi’an, People’s Republic of China; 5 Department of Pathology, The First Affiliated Hospital of Xi’an Jiaotong University, Xi’an, People’s Republic of China

**Keywords:** Exosomes, LncRNA-UCA1, Hypoxia, Bladder cancer

## Abstract

**Background:**

To overcome the hostile hypoxic microenvironment of solid tumors, tumor cells secrete a large number of non-coding RNA-containing exosomes that facilitate tumor development and metastasis. However, the precise mechanisms of tumor cell-derived exosomes during hypoxia are unknown. Here, we aim to clarify whether hypoxia affects tumor growth and progression by transferring long non-coding RNA-urothelial cancer-associated 1 (lncRNA-UCA1) enriched exosomes secreted from bladder cancer cells.

**Methods:**

We used bladder cancer 5637 cells with high expression of lncRNA-UCA1 as exosome-generating cells and bladder cancer UMUC2 cells with low expression of lncRNA-UCA1 as recipient cells. Exosomes derived from 5637 cells cultured under normoxic or hypoxic conditions were isolated and identified by transmission electron microscopy, nanoparticle tracking analysis and western blotting analysis. These exosomes were co-cultured with UMUC2 cells to evaluate cell proliferation, migration and invasion. We further investigated the roles of exosomal lncRNA-UCA1 derived from hypoxic 5637 cells by xenograft models. The availability of lncRNA-UCA1 in serum-derived exosomes as a biomarker for bladder cancer was also assessed.

**Results:**

We found that hypoxic exosomes derived from 5637 cells promoted cell proliferation, migration and invasion, and hypoxic exosomal RNAs could be internalized by three bladder cancer cell lines. Importantly, lncRNA-UCA1 was secreted in hypoxic 5637 cell-derived exosomes. Compared with normoxic exosomes, hypoxic exosomes derived from 5637 cells showed the higher expression levels of lncRNA-UCA1. Moreover, Hypoxic exosomal lncRNA-UCA1 could promote tumor growth and progression though epithelial-mesenchymal transition, in vitro and in vivo. In addition, the expression levels of lncRNA-UCA1 in the human serum-derived exosomes of bladder cancer patients were higher than that in the healthy controls.

**Conclusion:**

Together, our results demonstrate that hypoxic bladder cancer cells remodel tumor microenvironment to facilitate tumor growth and development though secreting the oncogenic lncRNA-UCA1-enriched exosomes and exosomal lncRNA-UCA1 in human serum has the possibility as a diagnostic biomarker for bladder cancer.

**Electronic supplementary material:**

The online version of this article (10.1186/s12943-017-0714-8) contains supplementary material, which is available to authorized users.

## Background

Intratumoral hypoxia has been widely acknowledged as one of the most fundamental tumor microenvironment stresses for solid tumors, which is unfavorable for the rapid expansion of tumors [1]. In this harsh microenvironment, tumor cells reshape their surrounding microenvironment not only to sustain survival and optimal growth but also to subsequently promote invasion and dissemination [2]. However, the mechanisms of cancer cells remodeling the tumor microenvironment to adapt hypoxia remain unknown. Small extracellular vesicles also known as exosomes can be released by tumor cells to promote tumor progression and metastasis, which represents an important and effective mechanism for improving communication between tumor cells and their microenvironment [3, 4]. Therefore, it is clear that a better comprehension of the microenvironment changes induced by tumor cell-derived exosomes could be useful for targeting both primary tumor growth and metastasis.

Tumor cell-derived exosomes as signal transporters modulate local and systemic tumor microenvironment by horizontal transferring bioactive factors such as proteins, RNAs, and DNAs [5]. Notably, RNAs are reported to be the predominant molecular cargos of tumor cell-derived exosomes, and many species of non-coding RNAs including microRNAs (miRNAs) [6], circular RNAs [7] and long non-coding RNAs (lncRNAs) [8] are also enriched in these exosomes, which can reflect the dysregulated non-coding RNAs profiles in tumor cells [9]. Furthermore, these functional non-coding RNAs delivered by exosomes to a recipient cell can regulate gene expression to promote primary tumor growth and local invasion and to favour the formation of premetastatic or metastatic niches [10–12]. Accumulating evidence has highlighted the exosomal non-coding RNAs can enter bloodstream or other body fluids, and these plasma/serum-derived or urinary-derived exosomal non-coding RNAs may serve as the early diagnostic or prognostic non-invasive biomarker for human cancers [13, 14]. To adapt hypoxic microenvironment, tumor cells can also stimulate exosomal secretion or regulate the content of exosomes, which accelerate tumor metastasis to more appropriate environment [15–17]. The hypoxic tumor cell-derived exosomal non-coding RNAs have also been reported to play significant roles in facilitating angiogenesis and dissemination [18, 19].

Urothelial cancer-associated 1 (UCA1) is first reported to be an oncogenic lncRNA which is highly overexpressed in bladder cancer tissues and promotes bladder cancer cell proliferation by regulating several different downstream targets or pathways, including cAMP response element-binding protein (CREB), chromatin remodeling factor (BRG1), phosphoinositide 3-kinase (PI3K), protein kinase B (AKT) and Wnt pathways [20–25]. Moreover, lncRNA-UCA1 can also promote EMT, migration, and invasion of bladder cancer cells though the hsa-miR-145–zinc finger E-box binding homeobox 1⁄2 (ZEB1⁄2)–fascin homologue 1 (FSCN1) pathway [26]. Importantly, UCA1 has also been identified as a hypoxia-responsive lncRNA that can promote the proliferation, migration, and invasion of bladder cancer cells under hypoxia [27]. However, the precise mechanism by which lncRNA-UCA1 modulates bladder tumor growth and progression under hypoxia has not been completely elucidated. Additionally, a recent study reported that lncRNA-UCA1 can be packed in exosomes secreted from breast cancer cells [28]. Therefore, from the results of these studies, we speculated that hypoxia reshapes the tumor microenvironment to enhance bladder tumor growth and progression by transferring exosomal lncRNA-UCA1.

Here, we demonstrated that hypoxic exosomes derived from bladder cancer cells promote cell proliferation, migration and invasion. Remarkably, hypoxia enhances exosome-mediated transferring of lncRNA-UCA1 and hypoxic exosomal lncRNA-UCA1 enhances tumor growth in vitro and in vivo. Furthermore, hypoxic exosomal lncRNA-UCA1 promotes tumor progression though epithelial-mesenchymal transition (EMT). In addition, the expression levels of exosomal lncRNA-UCA1 are higher in the bladder cancer patients’ serum than in healthy donors’ serum. Therefore, our studies elucidated the mechanism that hypoxia promotes bladder tumor growth and development and provided a potential diagnostic biomarker for bladder cancer.

## Results

### Identification of exosomes derived from bladder cancer cells cultured under normoxic and hypoxic conditions

**Fig. 1:**
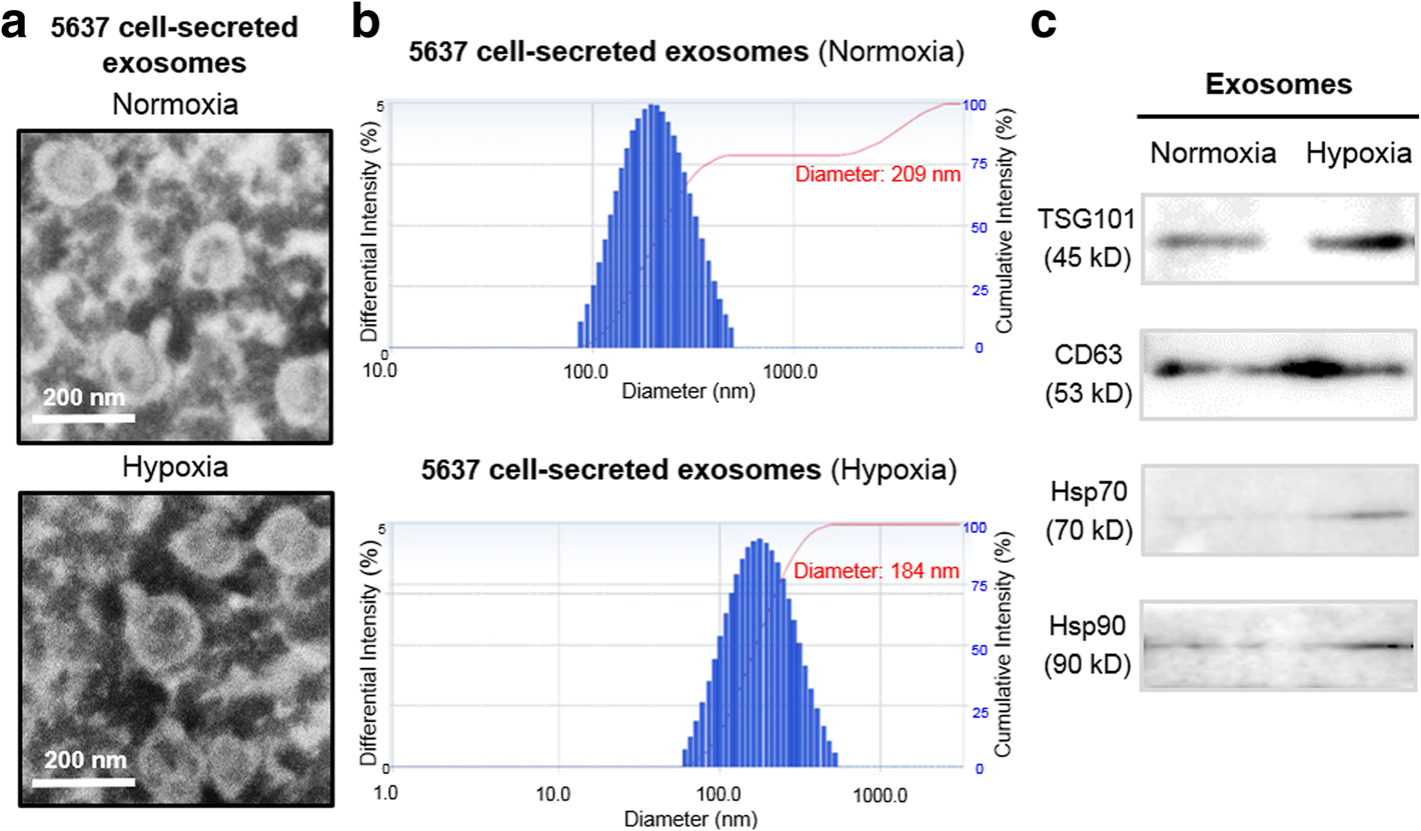
Characterization of normoxic and hypoxic exosomes derived from bladder cancer 5637 cells. **a** Transmission electron microscopy images of exosomes derived from normoxic and hypoxic 5637 cells. **b** Nanoparticle tracking analysis were analyzed the size distribution of normoxic and hypoxic exosomes derived from 5637 cells. **c** Western blotting analysis for exosomal markers TSG101, CD63, Hsp70 and Hsp90 of normoxic and hypoxic exosomes derived from 5637 cells; Equal amount of normoxic or hypoxic exosomes (500 ng) were used for western blotting analysis

Bladder cancer cell-derived exosomes were isolated from the conditioned media of 5637 cells cultured under normoxic or hypoxic conditions. The morphology of exosomes was analyzed by transmission electron microscopy (TEM). As shown in Fig. 1a, both normoxic and hypoxic exosomes showed typical lipid bilayer membrane-encapsulated nanoparticles and the size of these nanoparticles was 50–200 nm. Nanoparticle tracking analysis (NTA) exhibited that the average size of normoxic and hypoxic exosomes was 209 nm and 184 nm, respectively (Fig. 1b). There was a difference in the size distribution of exosomes between TEM and NTA analyses, and the main reason for such difference might be the aggregation behavior of exosomes in nanoparticle tracking analysis. Furthermore, we also characterized four exosomal protein markers of exosomes by western blotting analysis. As shown in Fig. 1c, the proteins of CD63, TSG101, Hsp70 and Hsp90 were positive expressed in normoxic or hypoxic exosomes. These results indicate that bladder cancer 5637 cells can secrete normoxic or hypoxic exosomes with common exosomal features.

### Hypoxic bladder cancer cell-derived exosomes promote bladder cancer cell proliferation, migration and invasion

**Fig. 2:**
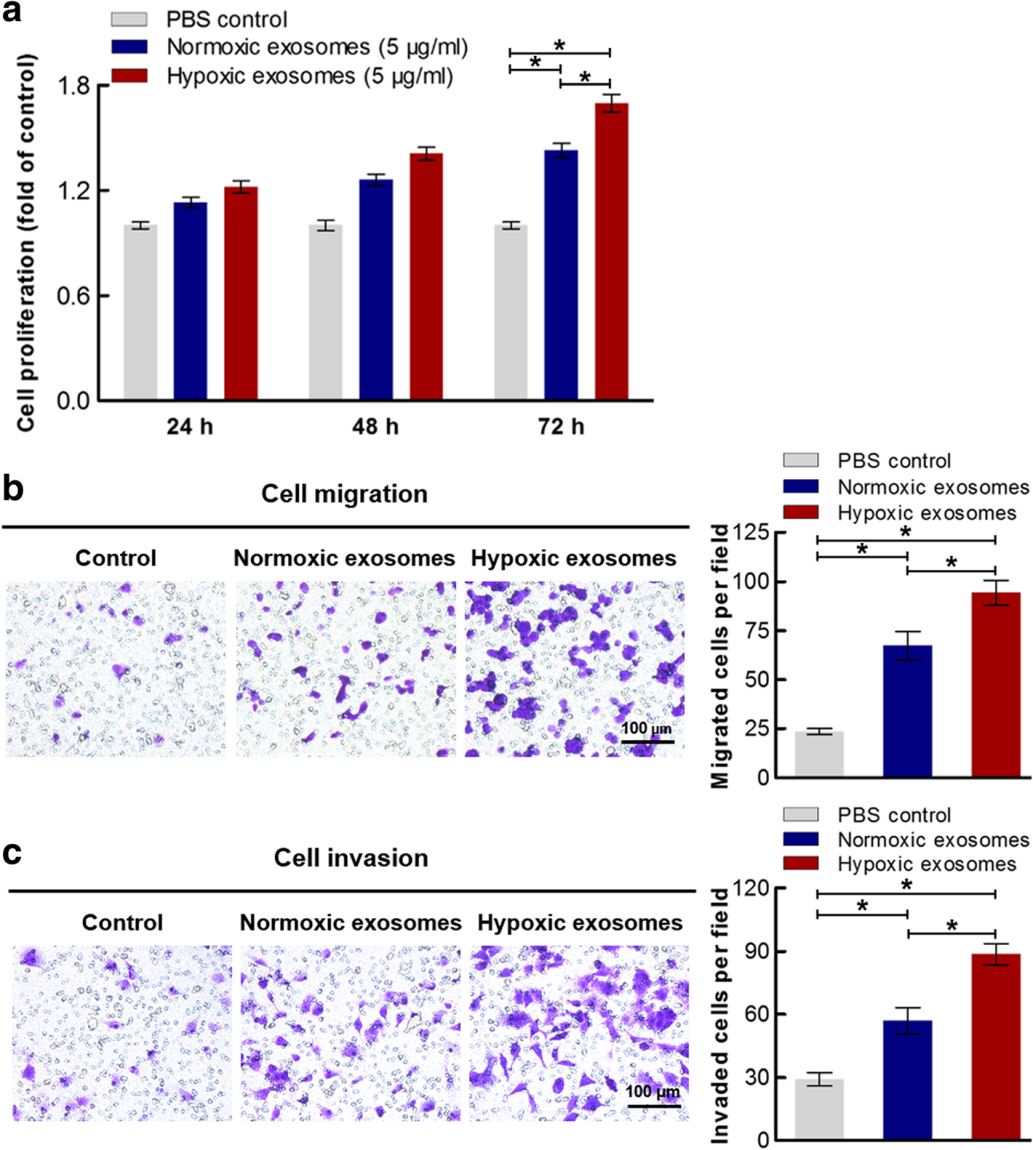
Roles of normoxic and hypoxic exosomes derived from 5637 cells. **a** Cell proliferation assay was performed in UMUC2 cells co-cultured with PBS, normoxic or hypoxic exosomes derived from 5637 cells (mean ± S.E.M., *P < 0.05, *n* = 3). **b** Migration assay was analyzed on the migratory potential of UMUC2 cells co-cultured with PBS, normoxic or hypoxic exosomes derived from 5637 cells (mean ± S.E.M., *P < 0.05, *n* = 3). **c** Invasion assay was analyzed on the invasiveness of UMUC2 cells co-cultured with PBS, normoxic or hypoxic exosomes derived from 5637 cells (mean ± S.E.M., *P < 0.05, *n* = 3)

To explore the biological roles of 5637 cell-derived normoxic and hypoxic exosomes, we next investigated the effects of normoxic and hypoxic exosomes on UMUC2 cell proliferation, migration and invasion. Compare with normoxic exosomes or PBS control, UMUC2 cells co-cultured with hypoxic exosomes showed higher cell viability (Fig. 2a). As shown in Fig. 2b, UMUC2 cells co-cultured with hypoxic exosomes exhibited higher motility than co-cultured with PBS control or normoxic exosomes. Similar to the cell migration results, enhanced the invasive potential of UMUC2 cells also occurred with 5637 cell-derived hypoxic exosomes (Fig. 2c). These results suggest that hypoxic bladder cancer cell-derived exosomes promote bladder cancer cell proliferation, migration and invasion in vitro.

### Hypoxia enhances exosome-mediated transferring of lncRNA-UCA1 into bladder cancer cells

**Fig. 3:**
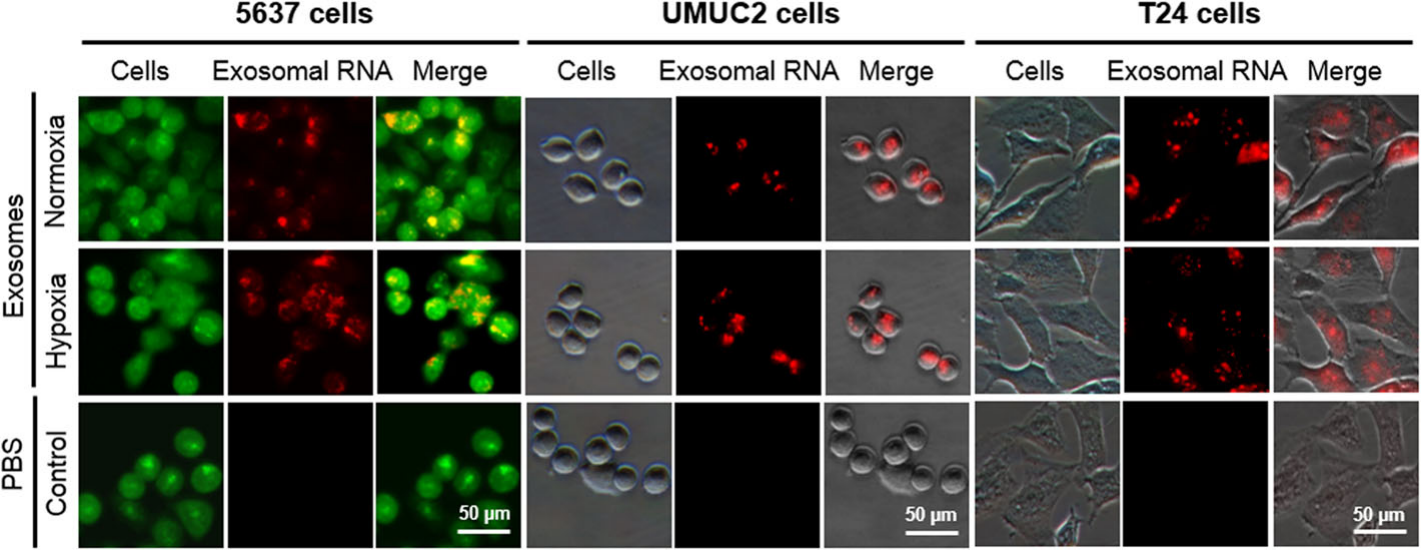
Internalization of normoxic and hypoxic exosomal RNAs derived from 5637 cells. Labelled-normoxic and hypoxic exosomal RNAs (*red* fluorescent dye) were uptake by 5637 (*green* fluorescent protein-labelled), UMUC2 and T24 cells

To investigate normoxic or hypoxic exosomal RNAs internalized by bladder cancer cell lines, 5637 cell-derived normoxic and hypoxic exosomal RNAs were labeled with Exo-Red dyes and incubated with three bladder cancer cell lines. As shown in Fig. 3, the labelled exosomal RNAs could be internalized by 5637 cells. Similar results were observed when adding the labelled exosomal RNAs to UMUC2 or T24 cells (Fig. 3).

**Fig. 4:**
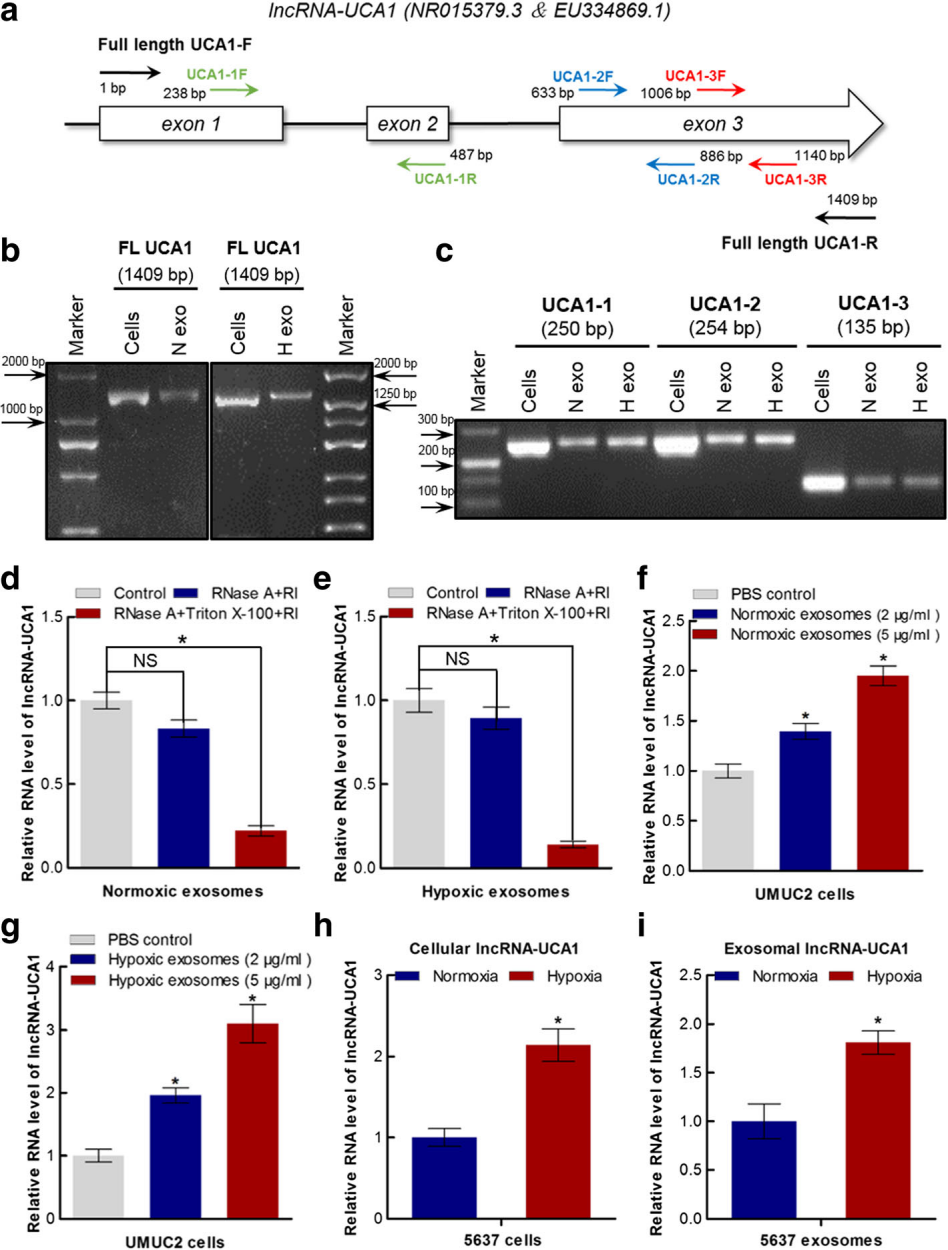
Identification of exosomal lncRNA-UCA1 in normoxic and hypoxic exosomes derived from 5637 cells. **a** Schematic representation of the UCA1 gene structure and the designed primers used for our study are shown in this schematic diagram. **b** and **c** Reverse transcription-PCR (RT-PCR) analysis of the full-length and fragments of lncRNA-UCA1 in normoxic and hypoxic exosomes derived from 5637 cells. **d** and **e** Quantitative real-time PCR (qRT-PCR) analysis of lncRNA-UCA1 expression in normoxic and hypoxic exosomes derived from 5637 cells. The samples were untreated with or treated with RNase A (10 μg/ml) and/or 0.3% Triton X-100 and then further mixed with of RNase inhibitor (mean ± S.E.M., *P < 0.05, *n* = 3﻿, NS, not significant). **f** and **g** qRT-PCR analysis of lncRNA-UCA1 expression in UMUC2 cells co-cultured with different concentrations of normoxic and hypoxic exosomes derived from 5637 cells (mean ± S.E.M., *P < 0.05, *n* = 3). **h** and **i** qRT-PCR analysis of lncRNA-UCA1 expression in normoxic and hypoxic 5637 cells and normoxic and hypoxic exosomes derived from 5637 cells (mean ± S.E.M., *P < 0.05, *n* = 3)

To further identify whether lncRNA-UCA1 is secreted in 5637 cell-derived normoxic and hypoxic exosomes, we first explored the existence pattern of lncRNA-UCA1 in exosomes. We designed primers to amplify the full-length transcript of UCA1 (Fig. 4a). Reverse transcription-PCR (RT-PCR) results showed that the full-length transcript of UCA1 (~1.4 kb) could be amplified from the normoxic and hypoxic exosomes (Fig. 4b). We also designed three primers for quantitative real-time PCR (qRT-PCR) to detect the expression levels of lncRNA-UCA1 in exosomes (Fig. 4a). According to the RT-PCR result, the UCA1–2 primers were used to detect exosomal lncRNA-UCA1 expression in our current study (Fig. 4c). We then determined whether lncRNA-UCA1 was indeed present within exosomes, which are provided a double-layer membrane against degradation by RNase. As expected, the expression levels of lncRNA-UCA1 in normoxic or hypoxic exosomes treated with RNase was similar to that in untreated control. Furthermore, the expression levels of lncRNA-UCA1 significantly decreased in normoxic or hypoxic exosomes treated with both RNase and Triton X-100 (Fig. 4d and e). These results indicate that the full-length transcript of UCA1 acts as an exosomal lncRNA transferred by bladder cancer cell-derived normoxic or hypoxic exosomes.

To further evaluate whether exosomal lncRNA-UCA1 was internalized by bladder cancer cells, we have also evaluated the relative UCA1 expression levels in different bladder cancer cell lines by RT-PCR and qRT-PCR. 5637 cells expressed relatively higher levels of UCA1, whereas UMUC2 cells expressed relatively lower levels of UCA1. Moreover, the expression levels of UCA1 could rarely be detected in UMUC2 cells by RT-PCR and qRT-PCR (Additional file 1: Figure S1a and b). Thus, UMUC2 cells were treated with different concentrations of 5637 cell-derived normoxic or hypoxic exosomes. The expressing levels of lncRNA-UCA1 in UMUC2 cells were upregulated in a dose-dependent manner compared with control cells treated with PBS (Fig. 4f and g). To clarify whether the expression levels of lncRNA-UCA1 in exosomes were induced by hypoxia, we detected intracellular or exosomal lncRNA-UCA1 expression levels by qRT-PCR. We found that hypoxia could induce the upregulation of lncRNA-UCA1 not only in bladder cancer cells but also in bladder cancer cell-derived exosomes (Fig. 4h and i). Collectively, these data indicate UCA1 may act as not only an intracellular hypoxia-responsive lncRNA but also an exosomal hypoxia-responsive lncRNA in bladder cancer.

### Hypoxic exosomal lncRNA-UCA1 promotes bladder cancer cell proliferation, migration and invasion

**Fig. 5:**
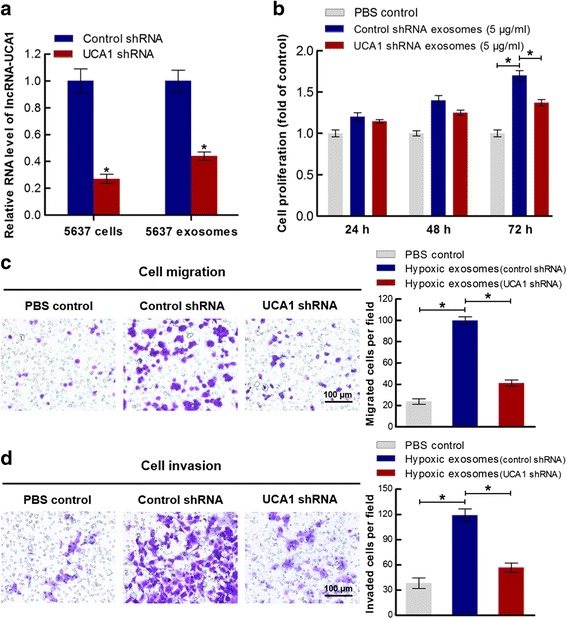
Hypoxic exosome-mediated bladder cancer cell proliferation, migration and invasion are dependent on lncRNA-UCA1. **a** Suppression efficiency of lncRNA-UCA1 shRNA in hypoxic 5637 cells or hypoxic exosomes derived from 5637 cells was detected by qRT-PCR (mean ± S.E.M., *P < 0.05, *n* = 3). **b** Cell proliferation assay was performed in UMUC2 cells co-cultured with PBS, hypoxic exosomes derived from 5637 cells transfected with control shRNA or UCA1 shRNA (mean ± S.E.M., *P < 0.05, *n* = 3). **c** Migration assay was analyzed on the migratory potential of UMUC2 cells co-cultured with PBS, hypoxic exosomes derived from 5637 cells transfected with control shRNA or UCA1 shRNA (mean ± S.E.M., *P < 0.05, *n* = 3). **d** Invasion assay was analyzed on the invasiveness of UMUC2 cells co-cultured with PBS, hypoxic exosomes derived from 5637 cells transfected with control shRNA or UCA1 shRNA (mean ± S.E.M., *P < 0.05, *n* = 3)

To investigate whether hypoxic exosome-mediated bladder cancer cell proliferation, migration and invasion are directly dependent on exosomal lncRNA-UCA1, we suppressed lncRNA-UCA1 expression in hypoxic 5637 cells by shRNA. The suppression efficiency of lncRNA-UCA1 shRNA in hypoxic 5637 cells was confirmed by qRT-PCR. In addition to affecting the expression levels of lncRNA-UCA1 in 5637 cells, lncRNA-UCA1 shRNA also inhibited the expression levels of lncRNA-UCA1 in 5637 cell-derived hypoxic exosomes (Fig. 5a). Furthermore, hypoxic control shRNA exosomes significantly promoted UMUC2 cell proliferation when compared to the PBS control group. Knockdown of lncRNA-UCA1 in hypoxic exosomes also led to a decrease in UMUC2 cell proliferation when compared to the hypoxic control shRNA exosomes group (Fig. 5b). Additionally, hypoxic control shRNA exosomes significantly increased UMUC2 cell mobility and invasion when compared to the PBS control group. Depletion of exosomal lncRNA-UCA1 in hypoxic exosomes decreased UMUC2 cell mobility and its depletion also downregulated the invasive potential of UMUC2 cells (Fig. 5c and d). Therefore, these results suggest that hypoxic exosomes regulate bladder cancer cell proliferation, migration and invasion in vitro, in part by exosomal lncRNA-UCA1.

### Hypoxic exosomal lncRNA-UCA1 facilitates bladder tumor growth

**Fig. 6:**
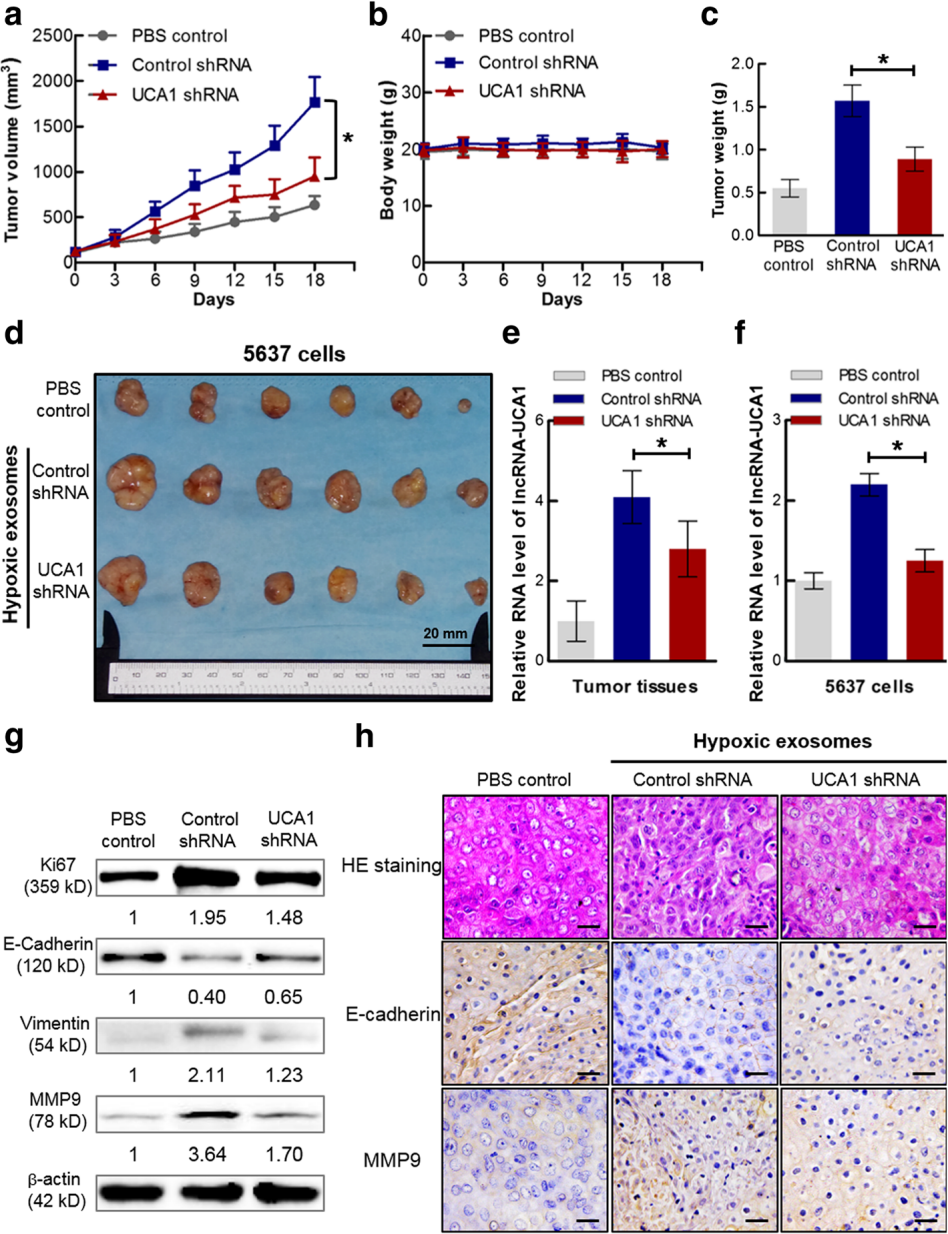
Hypoxic exosomal lncRNA-UCA1 promotes tumor growth and progression in vivo. **a** ~ **d** Effect of hypoxic exosomes derived from 5637 cells transfected with control shRNA or UCA1 shRNA on tumor growth in a xenograft model. Tumor volume and body weight of xenograft models were measured from day 0 to day 18, and final tumor weights were determined. (mean ± S.E.M., *P < 0.05, *n* = 6). **e** qRT-PCR analysis of lncRNA-UCA1 expression in xenograft tumor tissues treated with PBS or hypoxic exosomes derived from 5637 cells transfected with control shRNA or UCA1 shRNA (mean ± S.E.M., *P < 0.05, *n* = 3). **f** qRT-PCR analysis of lncRNA-UCA1 expression in 5637 cells co-cultured with PBS or hypoxic exosomes derived from 5637 cells transfected with control shRNA or UCA1 shRNA (mean ± S.E.M., *P < 0.05, *n* = 3). **g** Western blotting analysis of Ki67, E-cadherin, vimentin and MMP9 protein levels in 5637 cells co-cultured with PBS or hypoxic exosomes derived from 5637 cells transfected with control shRNA or UCA1 shRNA. **h** Hematoxylin and eosin-stained images and immunohistochemistry analysis of E-cadherin and MMP9 protein levels in xenograft tumor tissues (scale bar: 100 μm)

To further investigate the role of exosomal lncRNA-UCA1 in tumor growth in vivo, we next established a xenograft model (Additional file 2: Figure S2). After five weeks, mice were sacrificed and the xenograft tumor formation in the right flank. Furthermore, enlarged ipsilateral axillary lymph nodes were observed and removed for pathological examination (Additional file 3: Figure S3a). As shown in Fig. 6a and c, hypoxic control shRNA exosomes substantially increased tumor size and weight. Furthermore, depletion of lncRNA-UCA1 in hypoxic exosomes could abrogate the tumor growth (Fig. 6d). However, no metastases in lymph nodes were detected by immunohistochemistry using hematoxylin and eosin stain (Additional file 3: Figure S3b). Moreover, the primary tumor tissues of nude mice injected with hypoxic control shRNA exosomes had an increased expression of lncRNA-UCA1, and knockdown of lncRNA-UCA1 in hypoxic exosomes significantly decreased the expression of lncRNA-UCA1 in primary tumor tissues (Fig. 6e). These results indicate that hypoxic exosomes promote tumor growth is dependent on exosomal lncRNA-UCA1 in vivo.

### Hypoxic exosomal lncRNA-UCA1 promotes tumor progression though EMT

To elucidate the underlying mechanism of exosomal lncRNA-UCA1-mediated tumor growth and progression, 5637 cells were treated with hypoxic lncRNA-UCA1 shRNA exosomes or hypoxic control shRNA exosomes. As shown in Fig. 6f, the expressing levels of lncRNA-UCA1 in 5637 cells treated with hypoxic lncRNA-UCA1 shRNA exosomes were significantly downregulated. Western blotting analysis revealed the decreased expression levels of the proliferation marker Ki67 in hypoxic lncRNA-UCA1-depressing exosomes treated group (Fig. 6g). Furthermore, hypoxic lncRNA-UCA1-depressing exosomes also significantly increased the expression levels of E-cadherin, while markedly decreasing the expression levels of vimentin and MMP9 in vitro or in vivo (Fig. 6g and h). These findings indicate that hypoxic exosomal lncRNA-UCA1 promotes bladder tumor progression though EMT.

### Circulating exosomal lncRNA-UCA1 might act as a potential diagnostic biomarker for bladder cancer

**Fig. 7:**
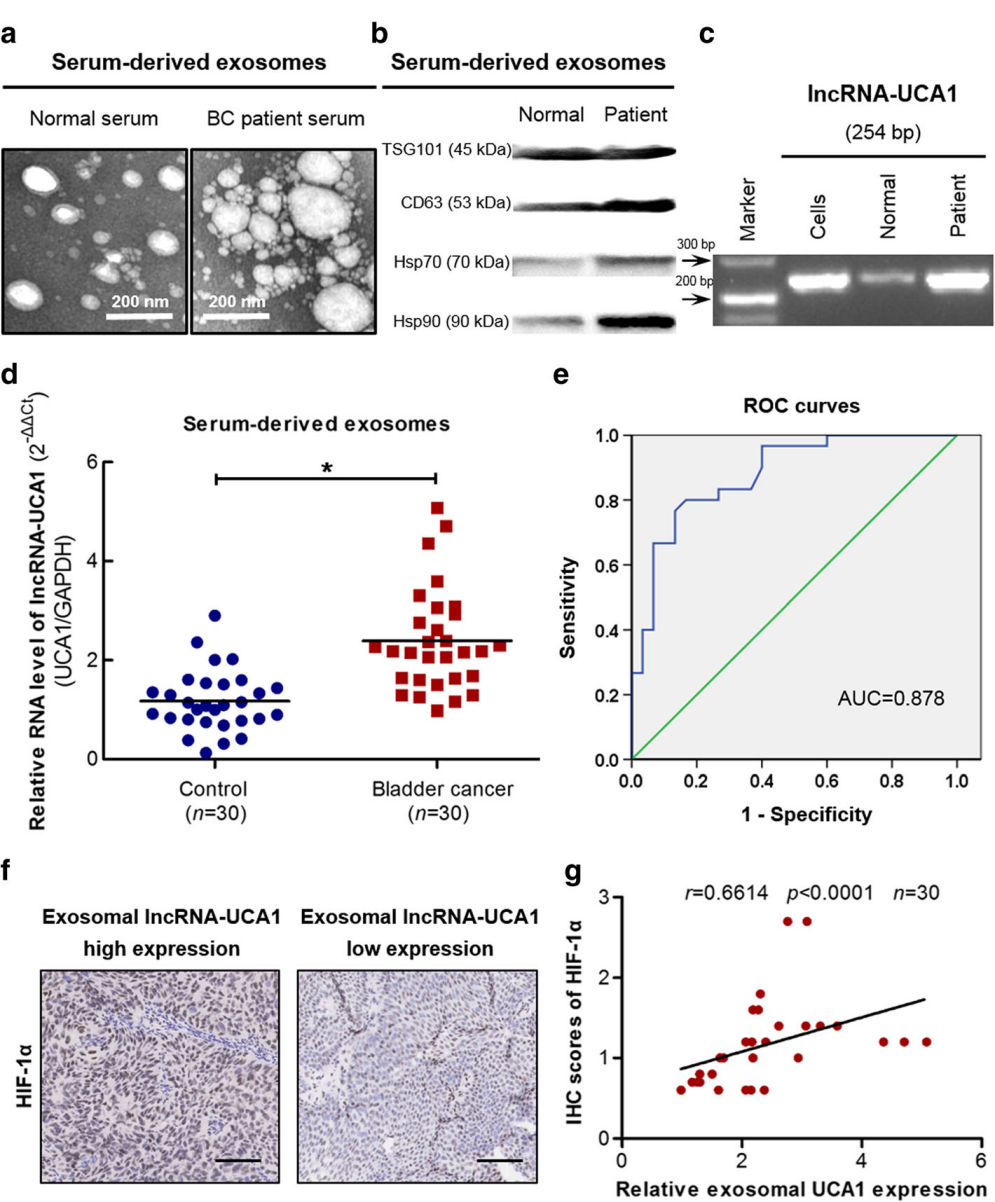
Circulating exosomal lncRNA-UCA1 serves as a potential non-invasive biomarker for bladder cancer diagnosis. **a** Transmission electron microscopy images of serum-derived exosomes from bladder cancer (BC) patients and healthy individuals. **b** Western blotting analysis for exosomal markers TSG101, CD63, Hsp70 and Hsp90 of serum-derived exosomes from bladder cancer patients and healthy individuals. **c** RT-PCR analysis of the fragments of lncRNA-UCA1 in 5637 cells and serum-derived exosomes from bladder cancer patients and healthy individuals. **d** qRT-PCR analysis of lncRNA-UCA1 expression in serum-derived exosomes from bladder cancer patients and healthy individuals (mean ± S.E.M., *P < 0.05) and data were normalized with GAPDH. **e** The ROC curve for the serum-derived exosomal lncRNA-UCA1 and GAPDH is an internal control. **f** Representative immunohistochemical staining images of higher expression of HIF-1α in bladder cancer samples with high exosomal lncRNA-UCA1 levels and lower expression of HIF-1α in samples with low exosomal lncRNA-UCA1 levels (scale bar: 100 μm). **g** Correlation analysis between immunohistochemistry (IHC) scores of HIF-1α and the expression levels of exosomal lncRNA-UCA1 in the serum of bladder cancer patients

Exosomal RNAs can be transferred from tumor cells to neighboring cells or cells at distant organs through entering the circulation [4]. Furthermore, hypoxia has been recognized as one of the hallmarks for solid tumors including bladder cancer [29]. Therefore, it is possible that exosomal lncRNA-UCA1 secreted by hypoxic bladder cancer cells can be detected in the circulation. To explore the circulating exosomal lncRNA-UCA1, we isolated exosomes from the serum of bladder cancer patients and matched healthy donors. TEM analysis revealed that the average size of isolated extracellular vesicles from bladder cancer patients or healthy individuals were exactly consistent with exosomes (50–200 nm in diameter, Fig. 7a). Moreover, western blotting analysis showed that the serum-derived exosomes from bladder cancer patients or healthy individuals were positive expressed for exosomal protein markers (Fig. 7b). To further identify lncRNA-UCA1 in serum-derived exosomes from bladder cancer patients or healthy individuals, we amplified the fragments of lncRNA-UCA1 in serum-derived exosomes. Indeed, the fragments of lncRNA-UCA1 from serum-derived exosomes of bladder cancer patients or healthy donors were determined by RT-PCR (Fig. 7c). We also found that the expression levels of exosomal lncRNA-UCA1 in the serum of bladder cancer patients were markedly higher than that in healthy control subjects, and the expression levels of exosomal lncRNA-UCA1 were normalized to ACTB (β-actin) or GAPDH (Fig. 7d and Additional file 4: Figure S4a). In addition, we used the ROC curve to evaluate the diagnostic value of exosomal lncRNA-UCA1 in serum. The ROC analysis demonstrated an area under curve (AUC) of 0.8783 (95% CI = 0.7926–0.964, *P* < 0.0001) for GAPDH better than ACTB (β-actin) (AUC: 0.7528, 95% CI = 0.6320–0.8736, *P* < 0.001) (Fig. 7e and Additional file 4: Figure S4b). The sensitivity and specificity were 80% and 83.33%, respectively (cut off value 1.603, GAPDH as an internal control). To further analyze the correlation between exosomal lncRNA-UCA1 and hypoxia marker HIF-1α in bladder cancer patients, HIF-1α expression was evaluated by immunohistochemical staining (Fig. 7f). As shown in Fig. 7g, exosomal lncRNA-UCA1 RNA levels in the serum of bladder cancer patients were positively correlated with HIF-1α expression. Altogether, these results indicate that exosomal lncRNA-UCA1 in serum may serve as a potential diagnostic biomarker for bladder cancer.

## Discussion

Hypoxic microenvironments of most prevalent malignancies exist in the interior away from their blood vessels. In these regions, cancer cells remodel their unfavorable microenvironment for tumor growth and metastatic dissemination, which usually cause resistance to cancer therapy [30]. Hence, it is necessary to explore the precise mechanism that how cancer cells reshape their surrounding microenvironment under hypoxic conditions. Several studies have described that exosomal proteins and miRNAs derived from hypoxic tumor cells are transferred or delivered to modulate biological function and cell signaling of recipient cells [19, 31, 32]. Apart from unique proteins or miRNAs, it is of interest to further investigate if hypoxia might promote tumor progression through transferring other species of exosomal non-coding RNA. In this study, we found that hypoxic exosome-mediated bladder cancer cell proliferation, migration and invasion are dependent on lncRNA-UCA1. Hypoxia enhances exosome-mediated shuttling of lncRNA-UCA1 into bladder cancer cells and hypoxic exosomal lncRNA-UCA1 also promotes tumor growth and progression in vitro and in vivo. Therefore, our data suggest that oncogenic lncRNA-UCA1 is an important content of hypoxic bladder cancer cell-derived exosomes to promote primary tumor growth and progression.

**Fig. 8:**
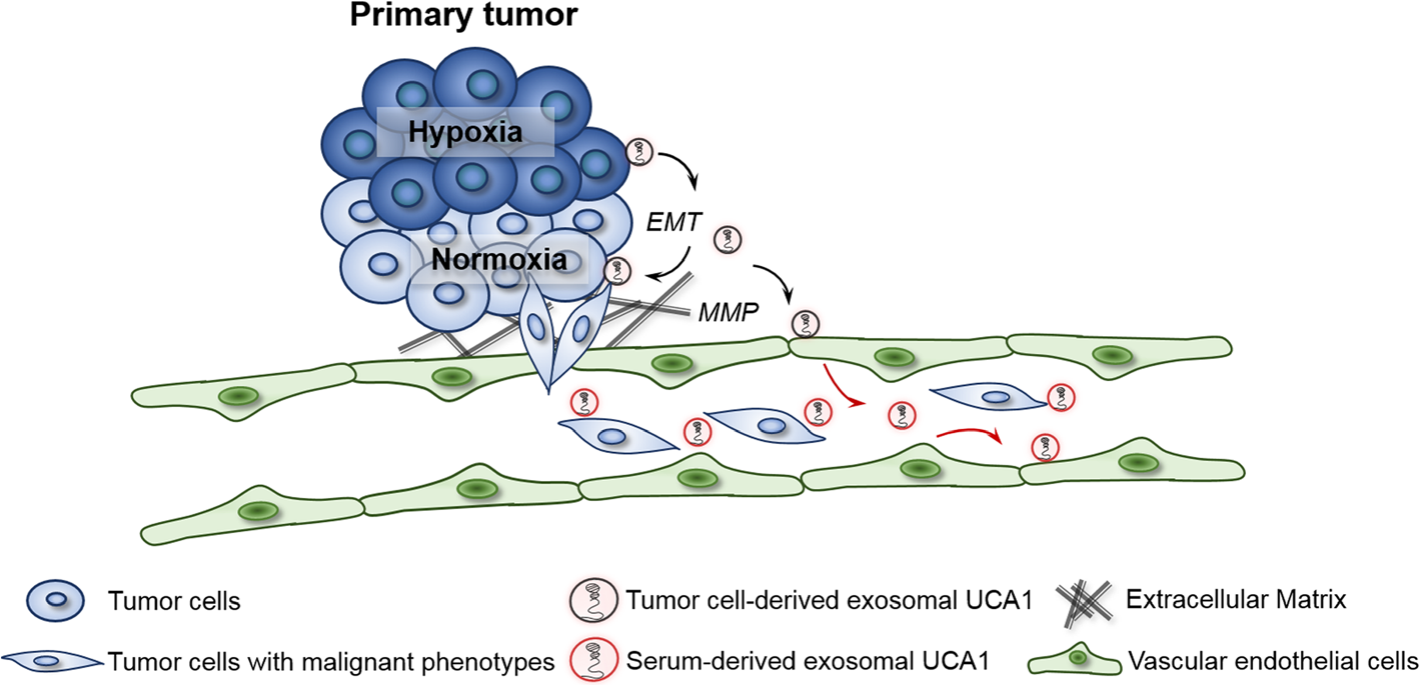
Schematic diagram of hypoxic exosomal lncRNA-UCA1-mediated bladder tumor growth and progression. Exosomal lncRNA-UCA1 derived from hypoxic bladder cancer cells promotes more aggressive phenotype of the neighbour cancer cells under normoxia and drives bladder tumor growth and development via EMT and MMP. Moreover, exosomal lncRNA-UCA1 in serum may serve as a potential non-invasive biomarker for bladder cancer diagnosis

Hypoxia has been reported to activate EMT for cancer invasion and dissemination [33]. Of note, our previous and other studies have shown that lncRNA-UCA1 functions as an inducer of EMT to promote cancer cells migration and invasion [26, 34]. Therefore, we speculate that hypoxia within the primary tumor may promote lncRNA-UCA1 transferred in exosomes to induce EMT. Our results are consistent with this hypothesis, and we found that knockdown of lncRNA-UCA1 in hypoxic exosomes increased E-cadherin expression, while decreasing the expression levels of vimentin and MMP9 in bladder cancer cells or xenograft tumor tissues. Together, these data provide evidence that hypoxia promotes exosomes transferred lncRNA-UCA1 though triggering EMT to cause the more malignant phenotypes of recipient cancer cells (Fig. 8).

Hypoxia can induce the expression of several cancer-related lncRNAs in cancer cells [35–37]. Importantly, our previous study found that lncRNA-UCA1 is also a hypoxia-responsive lncRNA [27]. Here, we uncovered that hypoxia induces the upregulation of lncRNA-UCA1 not only in bladder cancer cells but also in bladder cancer cell-derived exosomes. Our findings indicate that the mechanism of hypoxia-mediated shuttling of lncRNA-UCA1 into bladder cancer exosomes may depend on the upregulation of intracellular lncRNA-UCA1 induced by hypoxia. Therefore, hypoxia is verified to regulate the content of exosomes derived from bladder cancer cells.

Non-coding RNAs are encapsulated in exosomes to protect RNA from degradation by ribonuclease and hence these exosomal non-coding RNAs are particularly stable in body fluids [38, 39]. Here, we validated that exosomes provided a protective membrane for lncRNA-UCA1 against RNase degradation. Remarkably, some lncRNAs have been described to exert their biological functions though long RNA forms or being processed into shorter RNA forms [40]. Therefore, the integrity of lncRNA-UCA1 in tumor cell-derived exosomes needs to be identified. Our study indicated that the full-length form of lncRNA-UCA1 (~1.4 kb) is detected in bladder cancer cell-derived normoxic and hypoxic exosomes. This ~1.4 kb transcript is the major form of lncRNA-UCA1 in human cancer cells and it can promote tumor growth and progression [41]. Our results suggest that the ~1.4 kb transcript of lncRNA-UCA1 plays an oncogenic role not only in bladder cancer cells but also in extracellular milieu.

When exosomes released from tumor cells, these may enter the circulation. To date, exosomal non-coding RNAs have been detected in plasma, serum, urine, and other body fluids [13]. The expression levels of some exosomal lncRNAs correlate with the clinical classification and prognosis of human cancers and may be used for cancer biomarkers [42–44]. Our results were consistent with previous reports showing that exosomal lncRNA-UCA1 levels in the serum of bladder cancer patients are higher when compared to normal subjects. These results provide novel evidence that circulating exosomal lncRNA-UCA1 may serve as a potential biomarker for bladder cancer diagnosis. Nevertheless, the correlation between exosomal lncRNA-UCA1 expression in serum and tumor grade, stage and poor prognosis of bladder cancer patients remains unknown, and the diagnostic or prognostic values of circulating exosomal lncRNA-UCA1 in bladder cancers still needs further investigation in a large-scale clinical study.

In summary, our study demonstrated that hypoxia reshapes tumor microenvironment to promote bladder tumor growth and aggressiveness though releasing lncRNA-UCA1-containing exosomes and exosomal lncRNA-UCA1 in human serum may be considered as a potential diagnostic biomarker for bladder cancer.

## Methods

### Cell culture and hypoxia exposure

Human bladder cancer 5637, UMUC2 and T24 cells were grown in RPMI-1640 medium (Gibco, Grand Island, NY, USA) with 10% bovine calf serum and maintained at 37 °C under a humidified 5% CO_2_ atmosphere. For hypoxic experiments, hypoxia was achieved using an oxygen control incubator (Heal Force, Shanghai, China), which was flushed with a mixture of 1% O_2_, 94% N_2_, and 5% CO_2_. The cells were cultured for 24–72 h under hypoxic conditions.

### Clinical specimens

Human serum samples were collected from the First Affiliated Hospital of Xi’an Jiaotong University (Xi’an, Shaanxi, P.R. China) and obtained informed consent from 30 patients diagnosed bladder cancer and 30 healthy volunteers without any malignancy. The study was approved by the Ethics Committee of the First Affiliated Hospital of Xi’an Jiaotong University. The clinical pathological characteristics of bladder cancer patients are listed in Additional file 5: Table S1.

### Isolation of bladder cancer cell-derived exosomes

Bladder cancer 5637 cells were cultured for 48–72 h in advanced RPMI-1640 medium (Gibco) without supplements under normoxic or hypoxic conditions. Exosomes were isolated from the supernatant of 5637 cells by differential centrifugations as previously described [45]. The media were harvested and centrifuged at 300×g for 10 min at 4 °C. The supernatant was further centrifuged at 16,500×g for 20 min at 4 °C and filtered through a 0.22 μm filter. Exosomes were then pelleted by ultracentrifugation at 120,000×g for 70 min at 4 °C. Exosome pellets were resuspended in 0.2 μm-filtered PBS.

### Isolation of human serum-derived exosomes

Exosomes were isolated from the human serum samples by ExoQuick solution according to the manufacturer’s instructions (System Biosciences, SBI, Mountain View, CA). Briefly, serum samples were centrifuged at 3000×g for 15 min at 4 °C, and the serum samples (250 μl) were mixed with ExoQuick solution (63 μl). The mixtures were then refrigerated at 4 °C overnight and centrifuged at 1500×g for 30 min at 4 °C. Exosome pellets were resuspended in 0.2 μm-filtered PBS.

### Transmission electron microscopy

Exosomes were adsorbed to a 400 mesh carbon-coated copper grids and stained with phosphotungstic acid solution. Morphologies of the samples were observed by a JEOL JEM-100SX transmission electron microscope (JEOL Ltd., Tokyo, Japan).

### Nanoparticle tracking analysis

The size distribution of exosomes was determined using a Delsa Nano Analyzer (DelsaNano, Beckman Coulter, Brea, CA, USA). The capture settings and analysis settings were performed manually according to the manufacturer’s instructions.

### Quantitative real-time PCR

Exosomes were incubated with RNase A (Takara, Dalian, China, final concentration of 10 μg/ml) at 37 °C for 10 min followed by addition of RNase inhibitor (Takara, final concentration of 1 U/μl). Cellular and exosomal RNAs were isolated using the TRIzol reagent (Invitrogen, Life Technologies, Carlsbad, CA, USA). First-strand cDNA was synthesized with random primers using a PrimeScript™ RT reagent Kit with gDNA Eraser (Takara). Quantitative real-time PCR was carried out using a FastStart Essential DNA Green Master (Roche, Nutley, NJ, USA) on a CFX96 real-time PCR System (Bio-Rad, Hercules, CA, USA), and the results were normalized with ATCB (β-actin) or GAPDH as an internal control. Primers are listed in Additional file 6: Table S2.

### Western blotting analysis

Exosomes or cells were lysed in RIPA buffer containing a complete protease inhibitor tablet (Roche). The protein concentration of lysates was normalized according to Bradford relative protein quantification and proteins were separated by SDS-PAGE and transferred onto a nitrocellulose membrane. The membranes were incubated with CD63, E-cadherin, Ki67, MMP9, TSG101 (Abcam, Hong Kong, China), β-actin, Hsp70, Hsp90, Vimentin (Cell Signaling Technology, CST, Danvers, MA, USA) primary antibodies. Protein expression was assessed by ECL chemiluminescent regents and the intensity of the protein bands was quantified by densitometry using ImageJ software (National Institutes of Health).

### Fluorescent dye -labelled exosomal RNAs

Exosomal RNAs were labeled with Exo-GLOW™ kits (SBI) according to the manufacturer’s instructions. Exosomes were stained at 37 °C for 10 min in 1 × PBS containing 10 × Exo-Red dye and bladder cancer cells were incubated with the labeled exosomes at 37 °C for 6–24 h. Images were collected with a Nikon Eclipse Ti-S fluorescence microscope (Nikon, Tokyo, Japan).

### Knockdown experiment of exosomal lncRNA-UCA1

For depletion of lncRNA-UCA1, 5637 cells were stable transfected with pRNAT-U6.1⁄UCA1 shRNA or pRNAT-U6.1⁄control shRNA plasmid. The following day, 5637 cells were cultured in advanced 1640 medium under hypoxic conditions for 24–48 h. The exosomes derived from 5637 cells were purified as described previously.

### Cell proliferation assay

Cell proliferation assay was carried out using the Cell Count Kit (7Sea Pharmatech Co., Ltd., Shanghai, China). Briefly, UMUC2 cells (4000 cells/well) were seeded in 96-well plates and incubated with exosomes (5 μg/ml) for a total of 72 h. Absorbance was measured at 450 nm using a PerkinElmer Enspire plate reader (PerkinElmer, Waltham, MA, USA).

### Migration and invasion assays

The invasion assay was carried out using 24-well Millicell chambers that were coated with Matrigel (BD Biosciences, San Jose, CA, USA). The migration assay was carried out in a similar fashion without the Matrigel coating. Cells were seeded in serum-free RPMI-1640 to the top chamber. Exosomes (10 μg/ml each well) were added to the bottom chambers and serum-free advanced 1640 medium with PBS as the control. After 24 h of incubation, UMUC2 cells on the upper membrane surface were wiped off using a cotton swab and the lower membrane surface was fixed with methanol, stained with crystal violet, and counted in five random fields.

### Animal experiments

Five-week-old female athymic nude mice were purchased from the Laboratory Animal Center of Xi’an Jiaotong University (Xi’an, Shaanxi, P.R. China). The 5637 cells (5 × 10^6^ cells per mouse) suspended in 200 μl of PBS were injected subcutaneously into the right flank of nude mice, and two weeks later, when the nude mice generate tumors with a size of 100 mm^3^, purified exosomes (10 μg) or PBS were then injected into the center of tumor sites. The tumor size and weight of nude mice were weighed and measured twice a week. After three weeks, the nude mice were sacrificed and their tumors tissues and lymph nodes were determined for histological examination.

### Immunohistochemistry

Primary tumors were fixed in formalin and embedded in paraffin, and then cut at 4 μm thickness. The sections were incubated with primary antibodies (E-cadherin, HIF-1α, MMP9, Abcam) at 4 °C overnight. After washing with PBS, the sections were then incubated with Poly HRP-conjugated anti-mouse or anti-rabbit IgG for 30 min, followed by DAB. Finally, the sections were counterstained with hematoxylin. The staining intensity of HIF-1α was assessed by two independent pathologists as no staining = 0, weak staining = 1, moderate staining = 2, and strong staining = 3. Tumor cells in five random fields were scored based on the percentage of nuclei with positive staining cells (0–100%). An overall immunohistochemistry score was calculated by multiplying the intensity score with the percentage of positive staining tumor cells.

### Statistical analysis

All statistical analyses were carried out using GraphPad Prism Software (GraphPad Software, Inc., San Diego, CA, USA). Statistical evaluations were determined using Student’s *t*-test (two-tailed) or Spearman correlation test. *P* value <0.05 was considered statistically significant. In vitro experiments were replicated at least three times.

## Additional files

Additional file 1: Figure S1. The expression levels of lncRNA-UCA1 in different bladder cancer cell lines. **a** LncRNA-UCA1 expression levels in 5637 and UMUC2 cells were analyzed by RT-PCR. ACTB (β-actin) was used as the internal control. **b** LncRNA-UCA1 expression levels in 5637 and UMUC2 cells were analyzed by qRT-PCR. ACTB (β-actin) was used as the internal control. (TIFF 411 kb)

Additional file 2: Figure S2. Schema of in vivo tumor growth assay. 5637 cells were injected subcutaneously into the right flank of nude mice, and two weeks later, when the nude mice generate tumors with a size of 100 mm^3^, purified exosomes (10 μg) or PBS were then injected into the center of tumor sites. After three weeks, the nude mice were sacrificed and their tumors tissues and lymph nodes were determined for histological examination. (TIFF 523 kb)

Additional file 3: Figure S3. **a** Enlargement of ipsilateral axillary lymph nodes in a xenograft model was observed at five weeks. **b** Hematoxylin and eosin-stained images of lymph nodes in the ipsilateral axillary (scale bar: 100 μm). (TIFF 1843 kb)

Additional file 4: Figure S4. **a** qRT-PCR analysis of lncRNA-UCA1 expression in serum-derived exosomes from bladder cancer patients and healthy individuals (mean ± S.E.M., *P < 0.05), and data were normalized with ACTB (β-actin). **b** The ROC curve for the serum-derived exosomal lncRNA-UCA1, and ACTB (β-actin) is an internal control. (TIFF 506 kb)

Additional file 5: Table S1. Clinical characteristics of patients with bladder cancer (*n* = 30). (DOC 51 kb)

Additional file 6: Table S2. Primer and shRNA list. (DOC 37 kb)
