# Supplementary figures and images for: Hypoxic exosomes facilitate bladder tumor growth and development through transferring long non-coding RNA-UCA1
Source: Mol Cancer. 2017 Aug 25;16:143. doi: 10.1186/s12943-017-0714-8 (PMC5574139; doi:10.1186/s12943-017-0714-8)

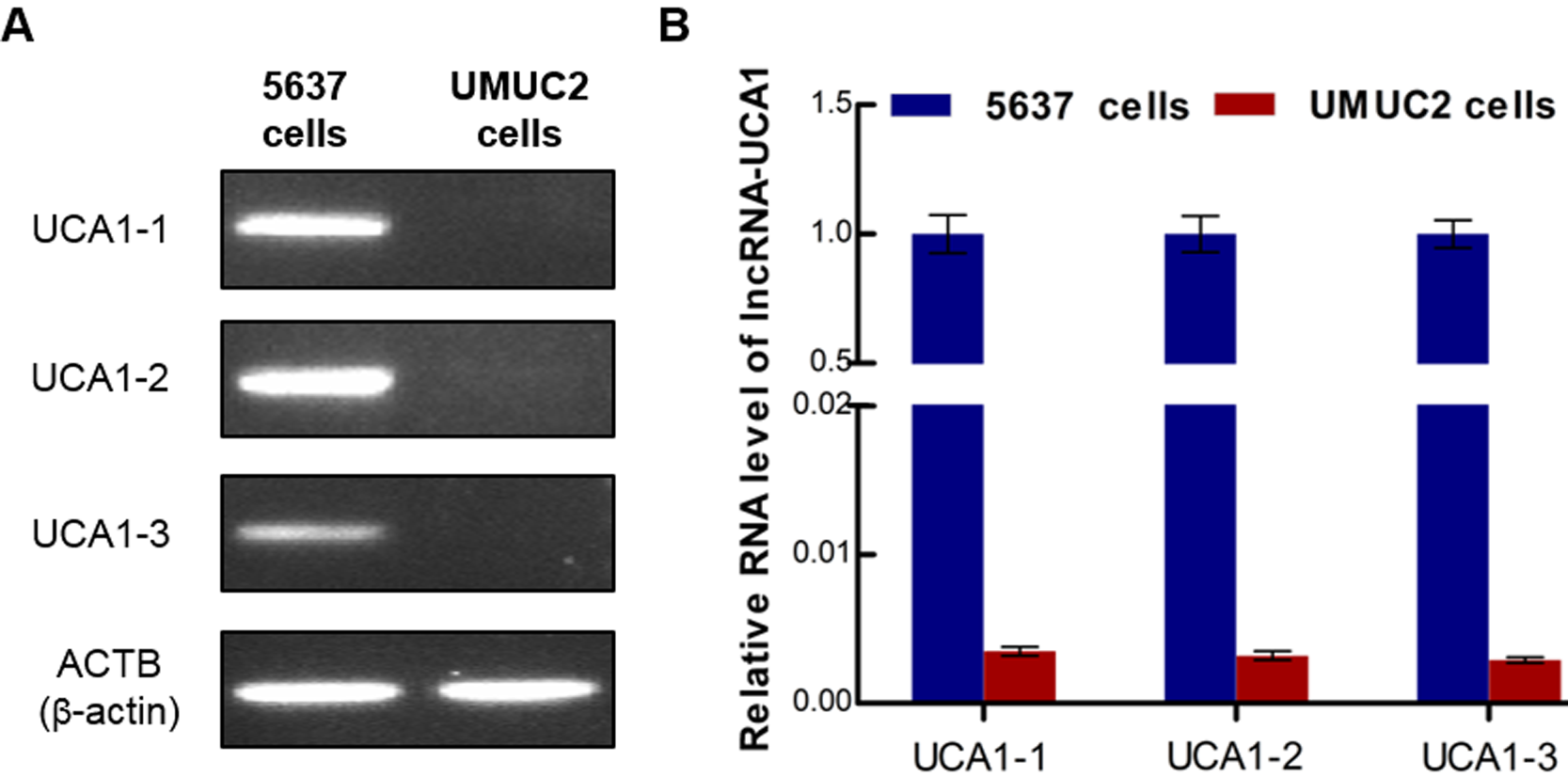

Supplement: Supplementary file 1 — The expression levels of lncRNA-UCA1 in different bladder cancer cell lines. a LncRNA-UCA1 expression levels in 5637 and UMUC2 cells were analyzed by RT-PCR. ACTB (β-actin) was used as the internal control. b LncRNA-UCA1 expression levels in 5637 and UMUC2 cells were analyzed by qRT-PCR. ACTB (β-actin) was used as the internal control. (TIFF 411 kb) [file 12943_2017_714_MOESM1_ESM.tif]

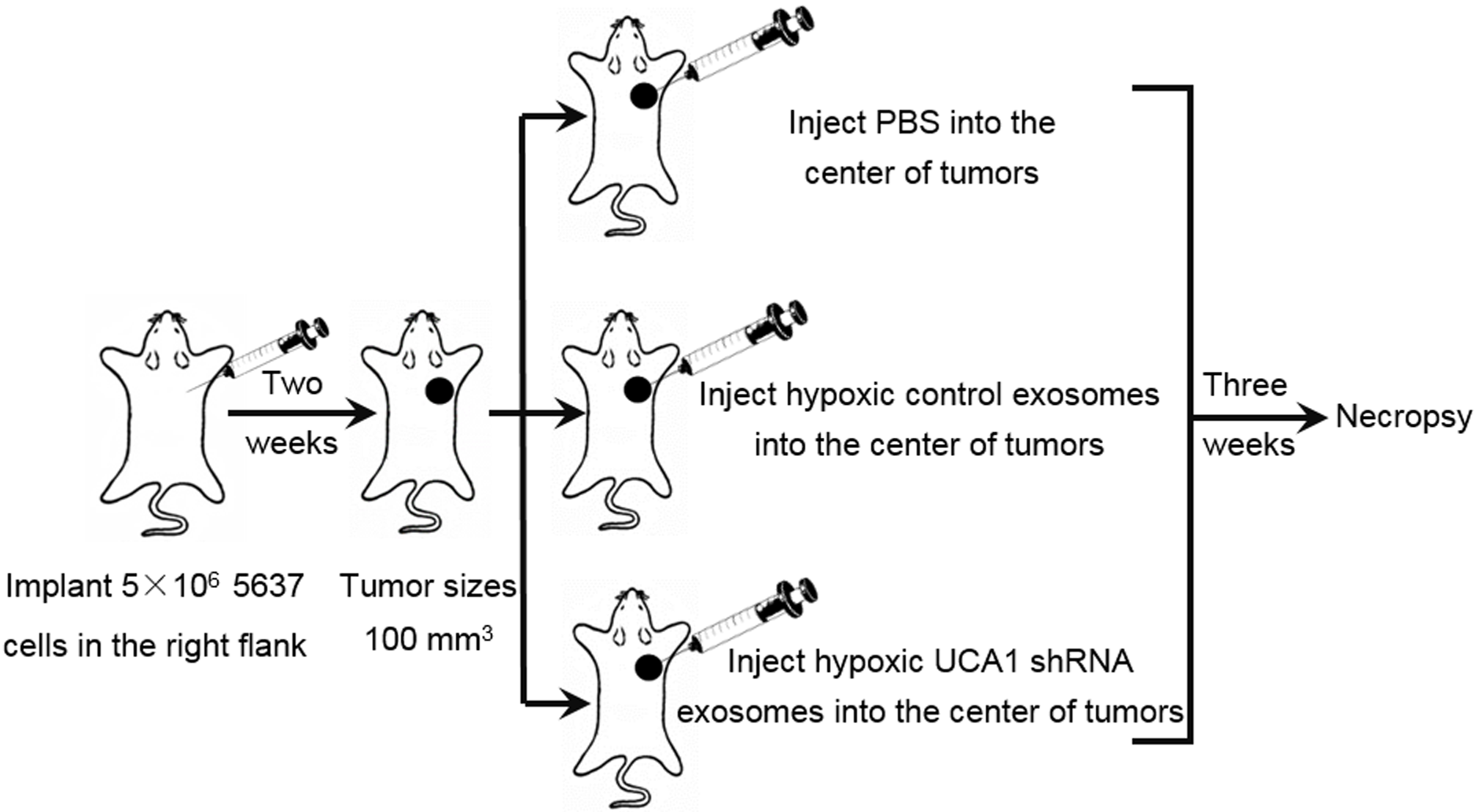

Supplement: Supplementary file 2 — Schema of in vivo tumor growth assay. 5637 cells were injected subcutaneously into the right flank of nude mice, and two weeks later, when the nude mice generate tumors with a size of 100 mm3, purified exosomes (10 μg) or PBS were then injected into the center of tumor sites. After three weeks, the nude mice were sacrificed and their tumors tissues and lymph nodes were determined for histological examination. (TIFF 523 kb) [file 12943_2017_714_MOESM2_ESM.tif]

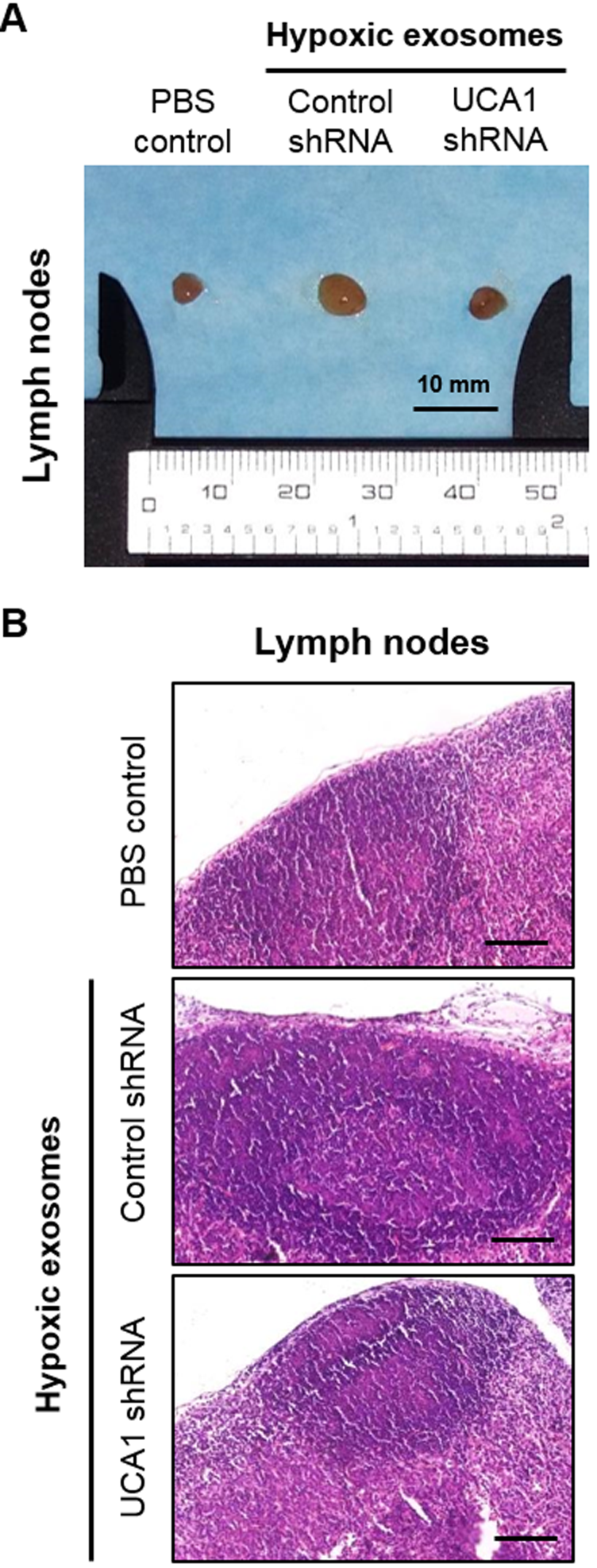

Supplement: Supplementary file 3 — a Enlargement of ipsilateral axillary lymph nodes in a xenograft model was observed at five weeks. b Hematoxylin and eosin-stained images of lymph nodes in the ipsilateral axillary (scale bar: 100 μm). (TIFF 1843 kb) [file 12943_2017_714_MOESM3_ESM.tif]

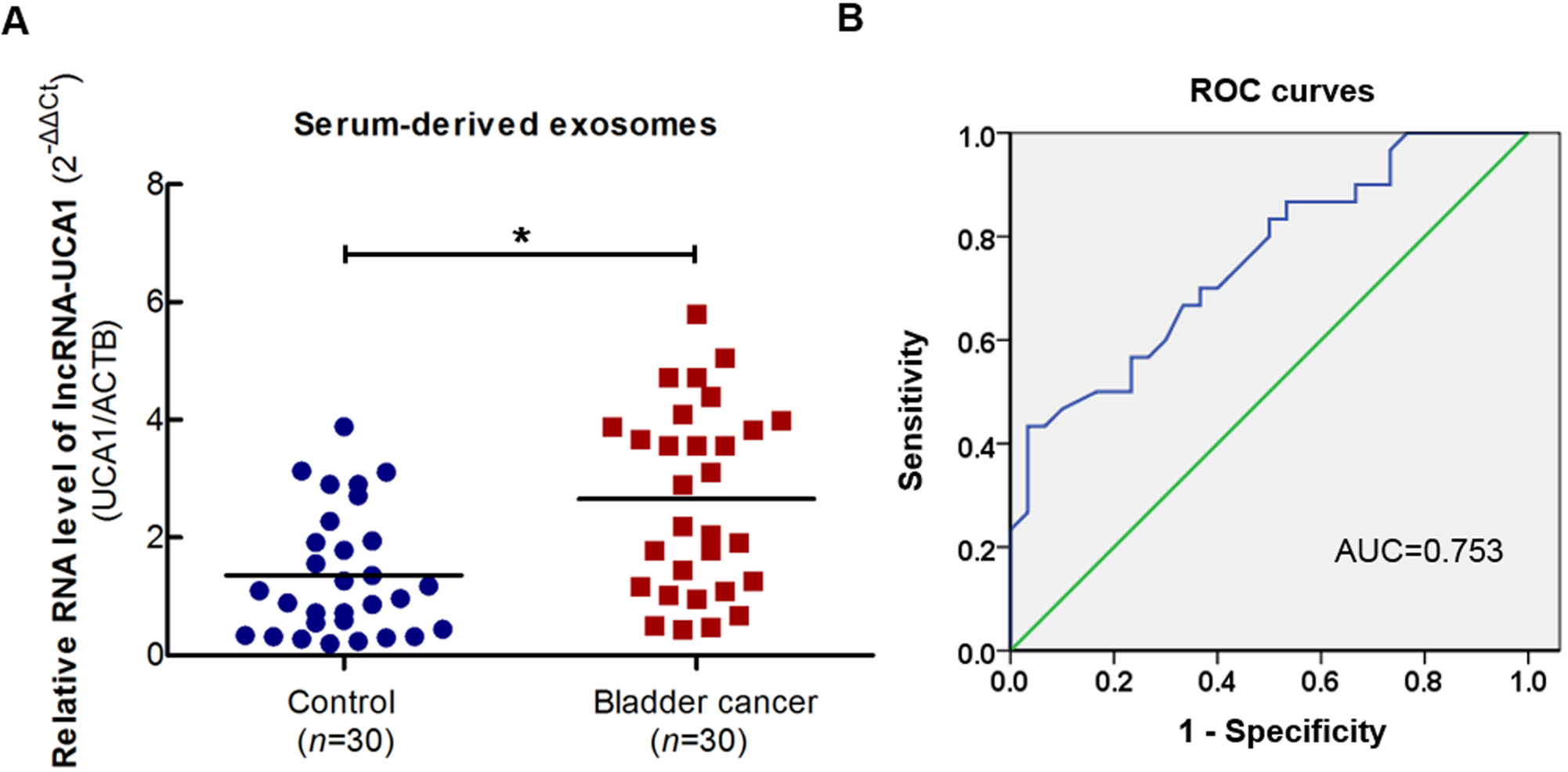

Supplement: Supplementary file 4 — a qRT-PCR analysis of lncRNA-UCA1 expression in serum-derived exosomes from bladder cancer patients and healthy individuals (mean ± S.E.M., *P < 0.05), and data were normalized with ACTB (β-actin). b The ROC curve for the serum-derived exosomal lncRNA-UCA1, and ACTB (β-actin) is an internal control. (TIFF 506 kb) [file 12943_2017_714_MOESM4_ESM.tif]
