# Supplementary material for: Hypoxic exosomes facilitate bladder tumor growth and development through transferring long non-coding RNA-UCA1
Source: Mol Cancer. 2017 Aug 25;16:143. doi: 10.1186/s12943-017-0714-8 (PMC5574139; doi:10.1186/s12943-017-0714-8)
Supplement: Supplementary file 5 — Clinical characteristics of patients with bladder cancer (n = 30). (DOC 51 kb) [file 12943_2017_714_MOESM5_ESM.doc]

Table S1

Clinical characteristics of patients with bladder cancer (*n*=30)

| **Sample number** | **Gender** | **Age (years)** | **Stage** | **Grade** |
| --- | --- | --- | --- | --- |
| 1 | Female | 56 | T1N0M0 | Low grade |
| 2 | Male | 72 | T1N0M0 | High grade |
| 3 | Female | 88 | T2N0M0 | High grade |
| 4 | Female | 78 | T3N0M0 | High grade |
| 5 | Male | 78 | T3N0M0 | High grade |
| 6 | Male | 84 | T1N0M0 | High grade |
| 7 | Female | 69 | T2N0M0 | High grade |
| 8 | Male | 66 | T2N0M0 | High grade |
| 9 | Female | 63 | T1N0M0 | Low grade |
| 10 | Male | 79 | T4N2M0 | High grade |
| 11 | Male | 84 | T2N0M0 | High grade |
| 12 | Male | 80 | T1N0M0 | Low grade |
| 13 | Female | 52 | T1N0M0 | Low grade |
| 14 | Male | 49 | T1N0M0 | Low grade |
| 15 | Male | 71 | T1N0M0 | High grade |
| 16 | Male | 52 | T1N0M0 | High grade |
| 17 | Male | 68 | T2N0M0 | High grade |
| 18 | Male | 53 | T1N0M0 | High grade |
| 19 | Male | 52 | T1N0M0 | Low grade |
| 20 | Male | 60 | T2N0M0 | High grade |
| 21 | Male | 51 | T1N0M0 | High grade |
| 22 | Male | 52 | T1N0M0 | High grade |
| 23 | Male | 80 | T1N0M1 | High grade |
| 24 | Female | 81 | T2N0M0 | Low grade |
| 25 | Female | 79 | T1N0M0 | Low grade |
| 26 | Male | 79 | T3N2M0 | High grade |
| 27 | Male | 88 | T2N0M0 | High grade |
| 28 | Male | 61 | T1N0M0 | Low grade |
| 29 | Male | 72 | T4N0M0 | High grade |
| 30 | Male | 71 | T2N0M0 | High grade |
