# Supplementary material for: Hypoxic exosomes facilitate bladder tumor growth and development through transferring long non-coding RNA-UCA1
Source: Mol Cancer. 2017 Aug 25;16:143. doi: 10.1186/s12943-017-0714-8 (PMC5574139; doi:10.1186/s12943-017-0714-8)
Supplement: Supplementary file 6 — Primer and shRNA list. (DOC 37 kb) [file 12943_2017_714_MOESM6_ESM.doc]

Table S2

Primer and shRNA list

| **Primers/shRNAs** | | **Sequences (5'–3')** | **Products legngth** |
| --- | --- | --- | --- |
| **Primers for quantitative real-time PCR or RT-PCR** | | | |
| UCA1-1F | | TACGGACATGCTTGACACTTG | 250 bp |
| UCA1-1R | | CGTAAGAGTTACCCGAAGCTC |  |
| UCA1-2F | | CTCTCCATTGGGTTCACCATTC | 254 bp |
| UCA1-2R | | GCGGCAGGTCTTAAGAGATGAG |  |
| UCA1-3F | | ATGTCCCAAGCCCTCTAAC | 135 bp |
| UCA1-3R | | ATGGTGTCCTCAAGCCTACT |  |
| Full length UCA1-F | | TGACATTCTTCTGGACAATGAGTCC | 1409 bp |
| Full length UCA1-R | | GGCATATTAGCTTTAATGTAGGTGGC |  |
| β-actin-F | | TCCCTGGAGAAGAGCTACGA | 194 bp |
| β-actin-R | | AGCACTGTGTTGGCGTACAG |  |
| GAPDH-F | | GGTGGTCTCCTCTGACTTCAA | 127 bp |
| GAPDH-R | | GTTGCTGTAGCCAAATTCGTTGT |  |
| **shRNAs for UCA1 silencing** | | | |
| shRNA control | gatccTTCTCCGAACGTGTCACGTttcaagagaACGTGACACGTTCGGAGAAttttttggaaa | | |
| UCA1 shRNA | gatccGTTAATCCAGGAGACAAAGAttcaagagaTCTTTGTCTCCTGGATTAACttttttggaaa | | |
